# Supplementary material for: Intergenerational effects of a casino-funded family transfer program on educational outcomes in an American Indian community
Source: Nat Commun. 2024 Sep 17;15:8168. doi: 10.1038/s41467-024-52428-w (PMC11408487; doi:10.1038/s41467-024-52428-w)
Supplement: Supplementary file 1 — Supplementary Information [file 41467_2024_52428_MOESM1_ESM.pdf]

Supplemental Table 1. Counts of American Indian and non-American Indian mothers (G2) by years of *duration* exposure, who later gave birth to children who have a recorded 3rd grade test score.

Years of Exposure < 18 years (for G2 mothers)	AI	Non-AI	Age at beginning of exposure (years)
none	252	1825	18+
<3	145	640	15 to 17
3 to <6	176	563	12 to 14
6 to <9	116	349	9 to 11
9 to <12	41	149	6 to 8
12+	10	23	<6

Supplemental Table 2: Test of pre-treatment parallel trends. Regression results predicting Third Grade **Math** Z-score for 1,931 children in Jackson, Swain, and Graham Counties, 2008-2017, as a function of American Indian race/ethnicity, mother's adult age minus 18 years in 1996 (i.e., number of pre-treatment years), and other covariates. Only children whose mothers were 18 years or older in 1996 are included in this falsification analysis.

**Variables**	**Model 2**		
	**coef**	**p-value**	**95% CI**
AI race/ethnicity (*AI*) (reference = non-AI)	-0.347	0.0003	[-0.534 -0.159]
Duration of pre-treatment exposure period of mother as an adult (pre_*treatment*)[1]	0.029	<.0001	[0.016 0.042]
Interaction of *AI* * pre_*treatment*	-0.011	0.545	[-0.048 0.025]
Offspring Age at test (years)	47.865	0.044	[1.230 94.499]
Offspring Age at test^2 (years)	-4.696	0.058	[-9.549 0.158]
Offspring Age at test^3 (years)	0.151	0.078	[-0.017 0.319]
Male sex at birth (reference = female)	0.015		[-0.066 0.096]
Intercept included	yes		

[1] This variable indicates number of pre-treatment years before cash transfer for American Indian participants; for non-American Indian participants, it represents an age control.

† We applied generalized estimating equation regressions[50] using maximum likelihood estimators, to predict the test score outcomes (PROC GENMOD in SAS). We used two-tailed tests for all statistical analyses.

Supplemental Table 3: Test of pre-treatment parallel trends. Regression results predicting Third Grade **Reading** Z-score for 1,915 children in Jackson, Swain, and Graham Counties, 2008-2017, as a function of American Indian race/ethnicity, mother's adult age minus 18 years in 1996 (i.e., number of pre-treatment years), and other covariates. Only children whose mothers were 18 years or older in 1996 are included in this falsification analysis.

**Variables**	**Model 2**		
	**coef**	**p-value**	**95% CI**
AI race/ethnicity (*AI*) (reference = non-AI)	-0.512	<.0001	[-0.704 -0.321]
Duration of pre-treatment exposure period of mother as an adult (pre_*treatment*)[1]	0.026	<.0001	[0.013 0.039]
Interaction of *AI* * pre_*treatment*	0.013	0.488	[-0.024 0.050]
Offspring Age at test (years)	78.697	0.002	[30.033 127.361]
Offspring Age at test^2 (years)	-7.869	0.002	[-12.942 -2.797]
Offspring Age at test^3 (years)	0.260	0.004	[0.084 0.435]
Male sex at birth (reference = female)	-0.115	0.006	[-0.197 -0.033]
Intercept included	yes		

[1] This variable indicates number of pre-treatment years before cash transfer for American Indian participants; for non-American Indian participants, it represents an age control.

[†] We applied generalized estimating equation regressions[50] using maximum likelihood estimators, to predict the test score outcomes (PROC GENMOD in SAS). We used two-tailed tests for all statistical analyses.

Supplemental Table 4: Restricting the analysis to a narrower range of mothers who received between 0 to 12 years of *duration* exposure by 1996. Regression results predicting Third Grade **Math** Z-score for 4,256 children in Jackson, Swain and Graham Counties, 2008-2017, as a function of American Indian race/ethnicity, duration of mother's exposure to family cash transfer as a child, and other covariates.

**Variables**	**Model 2**		
	**coef**	**p-value**	**95% CI**
AI race/ethnicity (*AI*) (reference = non-AI)	-0.417	<.0001	[-0.518 -0.316]
Duration of potential exposure of mother to family cash transfer in childhood (*duration*)[1]	-0.042	<.0001	[-0.051 -0.033]
Interaction of *AI * duration*	0.024	0.024	[0.003 0.046]
Offspring Age at test (years)	41.827	0.021	[6.414 77.239]
Offspring Age at test^2 (years)	-4.084	0.030	[-7.774 -0.393]
Offspring Age at test^3 (years)	0.131	0.046	[0.003 0.259]
Male sex at birth (reference = female)	0.025	0.382	[-0.030 0.079]
Intercept included	yes		

[1] This variable indicates number of pre-treatment years before cash transfer for American Indian participants; for non-American Indian participants, it represents an age control.

† We applied generalized estimating equation regressions[50] using maximum likelihood estimators, to predict the test score outcomes (PROC GENMOD in SAS). We used two-tailed tests for all statistical analyses.

Supplemental Table 5: Restricting the analysis to a narrower range of mothers who received between 0 to 12 years of *duration* exposure by 1996. Regression results predicting Third Grade **Reading** Z-score for 4,221 children in Jackson, Swain and Graham Counties, 2008-2017, as a function of American Indian race/ethnicity, duration of mother's exposure to family cash transfer as a child, and other covariates.

**Variables**	**Model 2**		
	**coef**	**p-value**	**95% CI**
AI race/ethnicity (*AI*) (reference = non-AI)	-0.483	<.0001	[-0.585 -0.380]
Duration of potential exposure of mother to family cash transfer in childhood (*duration*)[1]	-0.043	<.0001	[-0.053 -0.034]
Interaction of *AI * duration*	0.024	0.029	[0.003 0.046]
Offspring Age at test (years)	78.193	<.0001	[41.819 114.566]
Offspring Age at test^2 (years)	-7.825	<.0001	[-11.619 -4.031]
Offspring Age at test^3 (years)	0.258	0.0001	[0.127 0.390]
Male sex at birth (reference = female)	-0.108	0.0001	[-0.164 -0.053]
Intercept included	yes		

[1] This variable indicates number of years of possible receipt of cash transfer for American Indian participants; for non-American Indian participants, it represents an age control.

† We applied generalized estimating equation regressions[50] using maximum likelihood estimators, to predict the test score outcomes (PROC GENMOD in SAS). We used two-tailed tests for all statistical analyses.

Supplemental Table 6: Restricting the analysis to a narrower age range of G2 mothers 16 to 35 years at the time of G3's birth. Regression results predicting Third Grade **Math** Z-score for 3,977 children in Jackson, Swain and Graham Counties, 2008-2017, as a function of American Indian race/ethnicity, duration of mother's exposure to family cash transfer as a child, and other covariates.

**Variables**	**Model 2**		
	**coef**	**p-value**	**95% CI**
AI race/ethnicity (*AI*) (reference = non-AI)	-0.390	<.0001	[-0.495 -0.285]
Duration of potential exposure of mother to family cash transfer in childhood (*duration*)[1]	-0.038	<.0001	[-0.048 -0.029]
Interaction of *AI * duration*	0.021	0.056	[-0.0005 0.042]
Offspring Age at test (years)	47.954	0.010	[11.733 84.176]
Offspring Age at test^2 (years)	-4.728	0.014	[-8.500 -0.955]
Offspring Age at test^3 (years)	0.153	0.022	[0.022 0.284]
Male sex at birth (reference = female)	0.017	0.556	[-0.040 0.074]
Intercept included	yes		

[1] This variable indicates number of years of possible receipt of cash transfer for American Indian participants; for non-American Indian participants, it represents an age control.

[†] We applied generalized estimating equation regressions[50] using maximum likelihood estimators, to predict the test score outcomes (PROC GENMOD in SAS). We used two-tailed tests for all statistical analyses.

Supplemental Table 7: Restricting the analysis to a narrower age range of G2 mothers 16 to 35 years at the time of G3's birth. Regression results predicting Third Grade **Reading** Z-score for 3,944 children in Jackson, Swain and Graham Counties, 2008-2017, as a function of American Indian race/ethnicity, duration of mother's exposure to family cash transfer as a child, and other covariates.

**Variables**	**Model 2**		
	**coef**	**p-value**	**95% CI**
AI race/ethnicity (*AI*) (reference = non-AI)	-0.452	<.0001	[-0.558 -0.346]
Duration of potential exposure of mother to family cash transfer in childhood (*duration*)[1]	-0.040	<.0001	[-0.050 -0.031]
Interaction of *AI * duration*	0.022	0.048	[0.0002 0.044]
Offspring Age at test (years)	86.932	<.0001	[49.753 124.111]
Offspring Age at test^2 (years)	-8.738	<.0001	[-12.613 -4.863]
Offspring Age at test^3 (years)	0.290	<.0001	[0.156 0.424]
Male sex at birth (reference = female)	-0.094	0.001	[-0.151 -0.037]
Intercept included	yes		

[1] This variable indicates number of years of possible receipt of cash transfer for American Indian participants; for non-American Indian participants, it represents an age control.

[†] We applied generalized estimating equation regressions[50] using maximum likelihood estimators, to predict the test score outcomes (PROC GENMOD in SAS). We used two-tailed tests for all statistical analyses.

Supplemental Table 8: Control for test year (continuous). Regression results predicting Third Grade **Math** Z-score for 4,289 children in Jackson, Swain and Graham Counties, 2008-2017, as a function of American Indian race/ethnicity, duration of mother's exposure to family cash transfer as a child, and other covariates.

**Variables**	**Model 2**		
	**coef**	**p-value**	**95% CI**
AI race/ethnicity (*AI*) (reference = non-AI)	-0.423	<.0001	[-0.523 -0.323]
Duration of potential exposure of mother to family cash transfer in childhood (*duration*)[1]	-0.038	<.0001	[-0.048 -0.028]
Interaction of *AI* * *duration*	0.025	0.016	[0.005 0.045]
Test year (continuous)	-0.008	0.143	[-0.018 0.003]

[1] This variable indicates number of years of possible receipt of cash transfer for American Indian participants; for non-American Indian participants, it represents an age control.

‡ Child age and gender controls included in regression but not shown here (full results for covariates available in data repository).

† We applied generalized estimating equation regressions[50] using maximum likelihood estimators, to predict the test score outcomes (PROC GENMOD in SAS). We used two-tailed tests for all statistical analyses.

Supplemental Table 9: Control for test year (continuous). Regression results predicting Third Grade **Reading** Z-score for 4,254 children in Jackson, Swain and Graham Counties, 2008-2017, as a function of American Indian race/ethnicity, duration of mother's exposure to family cash transfer as a child, and other covariates.

**Variables**	**Model 2**		
	**coef**	**p-value**	**95% CI**
AI race/ethnicity (*AI*) (reference = non-AI)	-0.493	<.0001	[-0.594 -0.392]
Duration of potential exposure of mother to family cash transfer in childhood (*duration*)[1]	-0.043	<.0001	[-0.053 -0.033]
Interaction of *AI * duration*	0.028	0.007	[0.008 0.049]
Test year (continuous)	0.0006	0.910	[-0.010 0.012]

[1] This variable indicates number of years of possible receipt of cash transfer for American Indian participants; for non-American Indian participants, it represents an age control.

‡ Child age and gender controls included in regression but not shown here (full results for covariates available in data repository).

† We applied generalized estimating equation regressions[50] using maximum likelihood estimators, to predict the test score outcomes (PROC GENMOD in SAS). We used two-tailed tests for all statistical analyses.

Supplemental Table 10: Including Test Year Fixed Effects and restricting the analysis to a narrower age range of G2 mothers 16 to 35 years at the time of G3's birth. Regression results predicting Third Grade **Math** Z-score for 3,977 children in Jackson, Swain and Graham Counties, 2008-2017, as a function of American Indian race/ethnicity, duration of mother's exposure to family cash transfer as a child, and other covariates.

**Variables**	**Model 2**		
	**coef**	**p-value**	**95% CI**
AI race/ethnicity (*AI*) (reference = non-AI)	-0.376	<.0001	[-0.480 -0.273]
Duration of potential exposure of mother to family cash transfer in childhood (*duration*)[1]	-0.039	<.0001	[-0.049 -0.028]
Interaction of *AI* * *duration*	0.027	0.012	[0.006 0.048]
Test year fixed effects	Included; not shown		

[1] This variable indicates number of years of possible receipt of cash transfer for American Indian participants; for non-American Indian participants, it represents an age control.

‡ Child age and gender controls included in regression but not shown here (full results for covariates available in data repository).

† We applied generalized estimating equation regressions[50] using maximum likelihood estimators, to predict the test score outcomes (PROC GENMOD in SAS). We used two-tailed tests for all statistical analyses.

Supplemental Table 11: Including Test Year Fixed Effects and restricting the analysis to a narrower age range of G2 mothers 16 to 35 years at the time of G3's birth. Regression results predicting Third Grade **Reading** Z-score for 3,944 children in Jackson, Swain and Graham Counties, 2008-2017, as a function of American Indian race/ethnicity, duration of mother's exposure to family cash transfer as a child, and other covariates.

**Variables**	**Model 2**		
	**coef**	**p-value**	**95% CI**
AI race/ethnicity (*AI*) (reference = non-AI)	-0.443	<.0001	[-0.549 -0.337]
Duration of potential exposure of mother to family cash transfer in childhood (*duration*)[1]	-0.041	<.0001	[-0.051 -0.030]
Interaction of *AI * duration*	0.024	0.030	[0.002 0.046]
Test year fixed effects	Included; not shown		

[1] This variable indicates number of years of possible receipt of cash transfer for American Indian participants; for non-American Indian participants, it represents an age control.

‡ Child age and gender controls included in regression but not shown here (full results for covariates available in data repository).

† We applied generalized estimating equation regressions[50] using maximum likelihood estimators, to predict the test score outcomes (PROC GENMOD in SAS). We used two-tailed tests for all statistical analyses.

Supplemental Figure 1: Third Grade **Math** mean test scores normalized annually by Z- Score by test year and American Indian status.

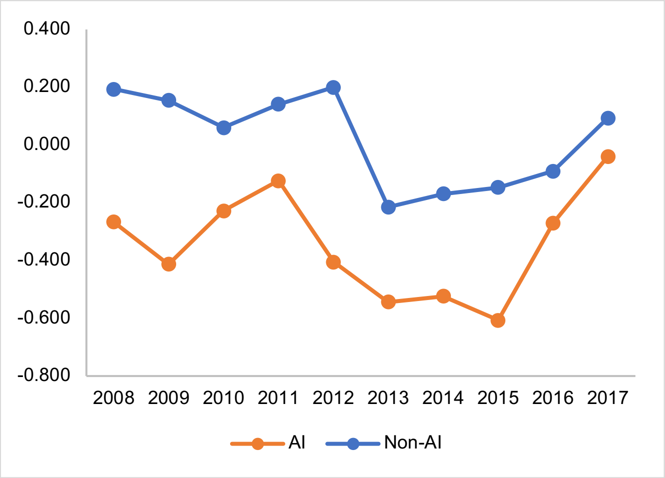

†Mean test scores for third grade math normalized annually by Z-score. American Indian scores are represented by the orange line. Non-American Indian scores are represented by the blue line.

Supplemental Figure 2: Third Grade **Reading** mean test scores normalized annually by Z-Score by test year and American Indian status.

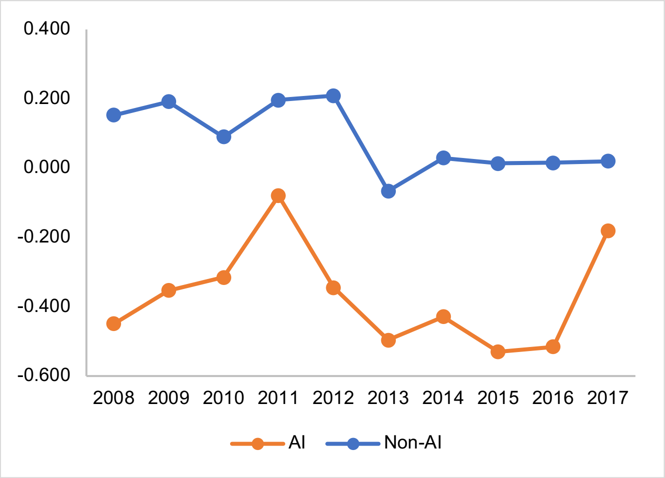

†Mean test scores for third grade reading normalized annually by Z-score. American Indian scores are represented by the orange line. Non-American Indian scores are represented by the blue line.
