# Supplementary material for: Intergenerational effects of a casino-funded family transfer program on educational outcomes in an American Indian community
Source: Nat Commun. 2024 Sep 17;15:8168. doi: 10.1038/s41467-024-52428-w (PMC11408487; doi:10.1038/s41467-024-52428-w)
Supplement: Supplementary file 2 — Reporting Summary [file 41467_2024_52428_MOESM2_ESM.pdf]

nature portfolio

Corresponding author(s): Tim Bruckner

Last updated by author(s): May 7, 2024

Reporting Summary

Nature Portfolio wishes to improve the reproducibility of the work that we publish. This form provides structure for consistency and transparency in reporting. For further information on Nature Portfolio policies, see our Editorial Policies and the Editorial Policy Checklist.

Statistics

For all statistical analyses, confirm that the following items are present in the figure legend, table legend, main text, or Methods section.

n/a	Confirmed	
☐	☒	The exact sample size (*n*) for each experimental group/condition, given as a discrete number and unit of measurement
☐	☒	A statement on whether measurements were taken from distinct samples or whether the same sample was measured repeatedly
☐	☒	The statistical test(s) used AND whether they are one- or two-sided<br>*Only common tests should be described solely by name; describe more complex techniques in the Methods section.*
☐	☒	A description of all covariates tested
☐	☒	A description of any assumptions or corrections, such as tests of normality and adjustment for multiple comparisons
☐	☒	A full description of the statistical parameters including central tendency (e.g. means) or other basic estimates (e.g. regression coefficient) AND variation (e.g. standard deviation) or associated estimates of uncertainty (e.g. confidence intervals)
☐	☒	For null hypothesis testing, the test statistic (e.g. *F*, *t*, *r*) with confidence intervals, effect sizes, degrees of freedom and *P* value noted<br>*Give P values as exact values whenever suitable.*
☒	☐	For Bayesian analysis, information on the choice of priors and Markov chain Monte Carlo settings
☒	☐	For hierarchical and complex designs, identification of the appropriate level for tests and full reporting of outcomes
☒	☐	Estimates of effect sizes (e.g. Cohen's *d*, Pearson's *r*), indicating how they were calculated

Our web collection on statistics for biologists contains articles on many of the points above.

Software and code

Policy information about availability of computer code

Data collection	N/A
Data analysis	Data analysis was conducted using SAS 9.4.

For manuscripts utilizing custom algorithms or software that are central to the research but not yet described in published literature, software must be made available to editors and reviewers. We strongly encourage code deposition in a community repository (e.g. GitHub). See the Nature Portfolio guidelines for submitting code & software for further information.

Data

Policy information about availability of data

All manuscripts must include a data availability statement. This statement should provide the following information, where applicable:

- Accession codes, unique identifiers, or web links for publicly available datasets
- A description of any restrictions on data availability
- For clinical datasets or third party data, please ensure that the statement adheres to our policy

The datasets generated during and/or analyzed during the current study are not publicly available due to the identifiable nature of the linked data, and the IRB human subjects regulations (Protocol: Pro00090215 with Duke University). Requests for data can be made to the NCERDC. In addition, to comply with open science requirements and that of NCERDC, group-level source data are provided with this paper, which includes the covariance matrix of the data analyzed along with a vector of means, standard deviations, and number of observations, separately by AI and non-AI participants. This information allows interested readers to re-create the regression analyses. The source data file also provides the summary data points used to create all figures.

Research involving human participants, their data, or biological material

Policy information about studies with human participants or human data. See also policy information about sex, gender (identity/presentation), and sexual orientation and race, ethnicity and racism.

Reporting on sex and gender	We compare children of American Indian and non-American Indian mothers. The test scores of children are grouped by subject matter and not sex or gender.
Reporting on race, ethnicity, or other socially relevant groupings	We use American Indian (AI) and non-American Indian (AI). We use these two categories because our exposure to a casino-cash transfer relates to American Indian race-ethnicity.
Population characteristics	AI mothers are more likely to have less than high school education (33.9%) compared to non-AI mothers (19.0%). AI mothers (37.7%) are less likely to be married than non-AI mothers (74.1%). AI mothers (23.4%) are more likely to use tobacco than non-AI mothers (21.2%). AI mothers are more likely to be less than 24 years of age than non- AI mothers (63.9% versus 44.7%). AI mothers also tend to have larger babies on average than non-AI mothers.
Recruitment	Administrative records of a child's birth and 3rd grade test scores were linked. Birth records occur for all reported births in North Carolina. Third grade test scores are collected for all third graders who take the state test at the end of the academic year.
Ethics oversight	The IRB human subjects regulations (Protocol: Pro00090215 with Duke University).

Note that full information on the approval of the study protocol must also be provided in the manuscript.

Field-specific reporting

Please select the one below that is the best fit for your research. If you are not sure, read the appropriate sections before making your selection.

☐ Life sciences ☒ Behavioural & social sciences ☐ Ecological, evolutionary & environmental sciences

For a reference copy of the document with all sections, see nature.com/documents/nr-reporting-summary-flat.pdf

Behavioural & social sciences study design

All studies must disclose on these points even when the disclosure is negative.

Study description	This is a quantitative study using linked birth records and educational outcomes data.
Research sample	The research sample includes AI and non-AI mothers and their children with 3rd grade test scores from Jackson, Swain, and Graham counties in North Carolina. The following text in the manuscript provides further details: "Using the North Carolina Education Research Data Center (NCERDC), we successfully linked to the North Carolina Birth file valid math (N = 4,289) and reading test scores (N = 4,254) for third grade public school students in this region, from 2008 to 2017. Whereas mean scores for non-AI children (N = 3,549) lie slightly above the state mean, those for AI children (N = 740) fall, on average, 0.39 standard deviations (SD) below the state mean (Figure 2).<br>Table 1 describes maternal and birth characteristics of the children with valid third grade test scores. AI mothers tend to report lower completed education, younger age at birth, and lower frequency of being married than do non-AI mothers. By contrast, the prevalence of preterm (<37 weeks completed gestational age at delivery) and/or low weight (<2,500 grams) delivery is lower among births to AI mothers (vs. non-AI mothers). These patterns appear consistent with the broader literature describing racial/ethnic differences, which indicates minimal bias in the NCERDC algorithm used to link birth records to third grade test scores."
Sampling strategy	The sample size includes 4,289 children with 3rd grad test scores and linked birth records. No sample size calculations were performed. The sample includes all children with a linked birth record and third grade test score for both AI and non-AI. The NCERDC reports an overall match rate >74%. The data include all records with a successful linkage.

Data collection	Data were collected by the North Carolina Education Research Data Center (NCERDC). More information on their data procedures can be found here https://childandfamilypolicy.duke.edu/north-carolina-education-research-data/.
Timing	Data include linked birth records and 3rd grade test scores for children in the 3rd grade between 2008 to 2017.
Data exclusions	Only records with linked birth records and 3rd grade education data were included.
Non-participation	The NCERDC reports a match rate of >74% beginning in 2008.
Randomization	Groups were defined by race/ethncity of the mother given the cash transfer was only provided to American Indian individuals.

Reporting for specific materials, systems and methods

We require information from authors about some types of materials, experimental systems and methods used in many studies. Here, indicate whether each material, system or method listed is relevant to your study. If you are not sure if a list item applies to your research, read the appropriate section before selecting a response.

Materials & experimental systems

n/a	Involved in the study
☒	☐ Antibodies
☒	☐ Eukaryotic cell lines
☒	☐ Palaeontology and archaeology
☒	☐ Animals and other organisms
☒	☐ Clinical data
☒	☐ Dual use research of concern
☒	☐ Plants

Methods

n/a	Involved in the study
☒	☐ ChIP-seq
☒	☐ Flow cytometry
☒	☐ MRI-based neuroimaging

Plants

Seed stocks	*Report on the source of all seed stocks or other plant material used. If applicable, state the seed stock centre and catalogue number. If plant specimens were collected from the field, describe the collection location, date and sampling procedures.*
Novel plant genotypes	*Describe the methods by which all novel plant genotypes were produced. This includes those generated by transgenic approaches, gene editing, chemical/radiation-based mutagenesis and hybridization. For transgenic lines, describe the transformation method, the number of independent lines analyzed and the generation upon which experiments were performed. For gene-edited lines, describe the editor used, the endogenous sequence targeted for editing, the targeting guide RNA sequence (if applicable) and how the editor was applied.*
Authentication	*Describe any authentication procedures for each seed stock used or novel genotype generated. Describe any experiments used to assess the effect of a mutation and, where applicable, how potential secondary effects (e.g. second site T-DNA insertions, mosiacism, off-target gene editing) were examined.*
